# Special Issue: “Bioinformatics and Omics Tools”

**DOI:** 10.3390/ijms241411625

**Published:** 2023-07-19

**Authors:** Rui Vitorino

**Affiliations:** iBiMED—Institute of Biomedicine, Department of Medical Sciences, University of Aveiro, 3810-193 Aveiro, Portugal; rvitorino@ua.pt

With the rapid introduction of high-throughput omics approaches such as genomics, transcriptomics, proteomics and metabolomics, the generation of large amounts of data has become a fundamental aspect of modern biological research [1]. These analyses generate enormous amounts of data on a daily basis, ranging from terabytes to petabytes. However, the integration of these multidimensional omics datasets into a biologically meaningful context is a major challenge due to differences in data size and nomenclature between different types of omics. In response to these challenges, terms such as integrated omics, multi-omics, poly-omics, trans-omics, and pan-omics have emerged, all referring to the integration of different omics datasets.

In the integration of omics data, one faces a variety of obstacles, including data cleaning, normalization, biomolecule identification, data dimensionality reduction, biological contextualization and statistical validation, as well as data storage, processing, sharing and archiving. The ultimate goal of these efforts is to gain a comprehensive understanding of biological systems through the lens of systems biology. Nevertheless, integration, interpretation, and obtaining meaningful insights present significant obstacles for current approaches.Overcoming these challenges requires the integration of large and disparate datasets into a cohesive framework, interpretation of the combined information, and derivation of meaningful insights from the integrated data. In addition, the reduction in cost and turnaround time for sample analyses have led to the generation of an ever-increasing number of omics datasets, including glycomics, lipidomics, microbiomics, and phenomics.

In this dynamic landscape, researchers in the interdisciplinary field of bioinformatics are at the forefront of addressing the complexities of integrating omics datasets. This Special Issue brings together a series of studies that discuss the current approaches, existing tools, and potential issues related to omics dataset integration. It highlights the need for standardized analytical pipelines that can be adopted by the global omics research community. These pipelines should enable researchers to effectively address the challenges of integrating, analyzing, and extracting meaningful insights from the vast and diverse omics datasets available to them.

The first study, entitled “PARM1 Drives Smooth Muscle Cell Proliferation in Pulmonary Arterial Hypertension via AKT/FOXO3A Axis”, focuses on pulmonary arterial hypertension (PAH), a debilitating disease with limited treatment options. Integrating bioinformatic analysis and experimental validation, the authors identified PARM1 as a potential therapeutic target for PAH. Their results highlight the role of PARM1 in promoting lung smooth muscle cell proliferation and provide insights into the underlying signaling pathway. This study is an example of how bioinformatics can aid in the discovery of novel targets for complex diseases [2].

The second study, entitled “Predicting Key Genes and Therapeutic Molecular Modeling to Explain the Association between *Porphyromonas gingivalis* (*P. gingivalis*) and Alzheimer’s Disease (AD)”, explores the intriguing link between *P. gingivalis* and Alzheimer’s disease. Using gene expression data and bioinformatics tools, the researchers identified common genes between *P. gingivalis* infection and AD and performed in silico molecular modeling to identify potential drug candidates targeting these genes. This study highlights the potential for integrating multiple omics datasets and computational approaches to elucidate complex disease associations and discover novel therapeutic opportunities [3].

In the third study, entitled “Quasispecies Fitness Partition to Characterize the Molecular Status of a Viral Population. Negative Effect of Early Ribavirin Discontinuation in a Chronically Infected HEV Patient”, the authors present a novel approach to analyzing viral quasispecies populations. By partitioning quasispecies into fractions based on fitness, they show how changes in these fractions can provide insights into the molecular changes that occur over time in a viral population. This study highlights the potential implications of these observations in the context of mutagenic antiviral treatments and illustrates the utility of bioinformatics in deciphering viral dynamics [4].

The study entitled “Coronary Artery Disease and Aortic Valve Stenosis: A Urine Proteomics Study” explores the use of urine proteomics as a noninvasive approach to identify biomarkers for coronary artery disease (CAD) and aortic valve stenosis (AVS). Through shotgun proteomics analysis, the researchers identified dysregulated proteins that could distinguish patients with CAD and/or AVS from healthy individuals. This study underscores the potential of omics technologies, particularly urine proteomics, for early disease detection and highlights promising biomarker candidates [5].

By establishing standardized approaches and tools, researchers can overcome the hurdles of integration, interpretation, and insight discovery. Such efforts can lead us to unlock the transformative potential of large-scale omics datasets, providing comprehensive and high-resolution insights into cellular systems. The resulting advancements contribute to an improved understanding of biological processes, events, and diseases through computational and informatics frameworks.

## References

[1] Cui M., Cheng C., Zhang L. (2022). High-throughput proteomics: A methodological mini-review. Lab. Investig..

[2] He Z., Chang T., Chen Y., Wang H., Dai L., Zeng H. (2023). PARM1 Drives Smooth Muscle Cell Proliferation in Pulmonary Arterial Hypertension via AKT/FOXO3A Axis. Int. J. Mol. Sci..

[3] Hamarsha A., Balachandran K., Sailan A.T., Nasruddin N.S. (2023). Predicting Key Genes and Therapeutic Molecular Modelling to Explain the Association between *Porphyromonas gingivalis* (*P. gingivalis*) and Alzheimer’s Disease (AD). Int. J. Mol. Sci..

[4] Gregori J., Colomer-Castell S., Campos C., Ibañez-Lligoña M., Garcia-Cehic D., Rando-Segura A., Adombie C.M., Pintó R., Guix S., Bosch A. (2022). Quasispecies Fitness Partition to Characterize the Molecular Status of a Viral Population. Negative Effect of Early Ribavirin Discontinuation in a Chronically Infected HEV Patient. Int. J. Mol. Sci..

[5] Perpétuo L., Barros A.S., Dalsuco J., Nogueira-Ferreira R., Resende-Gonçalves P., Falcão-Pires I., Ferreira R., Leite-Moreira A., Trindade F., Vitorino R. (2022). Coronary Artery Disease and Aortic Valve Stenosis: A Urine Proteomics Study. Int. J. Mol. Sci..

